# Anti-Inflammatory Mechanism Involved in Pomegranate-Mediated Prevention of Breast Cancer: the Role of NF-κB and Nrf2 Signaling Pathways

**DOI:** 10.3390/nu9050436

**Published:** 2017-04-28

**Authors:** Animesh Mandal, Deepak Bhatia, Anupam Bishayee

**Affiliations:** 1 Cancer Therapeutics and Chemoprevention Group, Department of Pharmaceutical Sciences, College of Pharmacy, Northeast Ohio Medical University, Rootstown, OH 44272, USA; animandal0@gmail.com; 2 Department of Pharmacogenomics, Bernard J. Dunn School of Pharmacy, Shenandoah University, Ashburn, VA 20147, USA; dbhatia@su.edu; 3 Department of Pharmaceutical Sciences, College of Pharmacy, Larkin University, Miami, FL 33169, USA

**Keywords:** anti-inflammatory effects, breast tumor, COX-2, DMBA, HSP90, NF-κB, Nrf2, *Punica granatum*

## Abstract

Pomegranate (*Punica granatum* L.), a nutrient-rich unique fruit, has been used for centuries for the prevention and treatment of various inflammation-driven diseases. Based on our previous study, a characterized pomegranate emulsion (PE) exhibited a striking inhibition of dimethylbenz(a)anthracene (DMBA)-initiated rat mammary tumorigenesis via antiproliferative and apoptosis-inducing mechanisms. The objective of the present work is to investigate the anti-inflammatory mechanism of action of PE during DMBA rat mammary carcinogenesis by evaluating the expression of cyclooxygenase-2 (COX-2), heat shock protein 90 (HSP90), nuclear factor-κB (NF-κB) and nuclear factor erythroid 2p45 (NF-E2)-related factor 2 (Nrf2). Mammary tumor samples were harvested from our previous chemopreventive study in which PE (0.2–5.0 g/kg) was found to reduce mammary tumorigenesis in a dose-dependent manner. The expressions of COX-2, HSP90, NF-κB, inhibitory κBα (IκBα) and Nrf2 were detected by immunohistochemical techniques. PE decreased the expression of COX-2 and HSP90, prevented the degradation of IκBα, hindered the translocation of NF-κB from cytosol to nucleus and increased the expression and nuclear translocation of Nrf2 during DMBA-induced mammary tumorigenesis. These findings, together with our previous results, indicate that PE-mediated prevention of DMBA-evoked mammary carcinogenesis may involve anti-inflammatory mechanisms through concurrent but differential regulation of two interrelated molecular pathways, namely NF-κB and Nrf2 signaling.

## 1. Introduction

Pomegranate (*Punica granatum* L.) is a nutrient-rich fruit which represents a reservoir of bioactive phytochemicals with exceptional medicinal values. The pomegranate is a plant native from the Himalayas to Iran and has been cultivated and naturalized throughout the world and in the United States, including Arizona, California and Texas. Pomegranate, known as “*a pharmacy unto itself*” has been used for centuries in various traditional and folk medicine for the treatment of a large number of ailments [1,2,3]. During the last decade, pomegranate fruit has been gaining a widespread reputation as a dietary supplement as well as a functional food due to emerging scientific evidence on potential health benefits, including prevention and/or treatment of cardiovascular ailments, neurological disorders, oncologic diseases, dental problems, inflammation, ulcer, arthritis, microbial infection, obesity, diabetes, acquired immune deficiency syndrome and erectile dysfunction [4,5,6,7,8,9]. Pomegranate fruit contains phytochemicals, including flavonoids (e.g., anthocyanins and catechins), flavonols (e.g., kaempferol and quercetin), flavones (e.g., apigenin and luteolin), conjugated fatty acids, hydrolyzable tannins and related compounds which are thought to be responsible for various biological and pharmacological activities [4,10,11,12,13,14]. Based on preclinical and clinical studies conducted by various laboratories worldwide, pomegranate-derived substances, such as juice, extracts and phytoconstituents exhibited cancer preventive and therapeutic effects against colon, liver, lung, prostate and skin cancer [4,15,16,17,18]. Various extracts, fractions and phytochemicals from pomegranate fruit, peel, seed and flower demonstrated cytotoxic, antiproliferative, proapoptotic, antiangiogenic, anti-invasive, and antimetastatic properties against estrogen receptor-positive and ‑negative breast cancer cells [19,20,21,22,23,24,25,26,27,28,29,30,31,32]. Pomegranate seed oil and fermented juice concentrate were found to suppress 7,12-dimethyl benz(a)anthracene (DMBA)-induced preneoplastic mammary gland lesions ex vivo in a mouse mammary organ culture model [33]. Oral administration of pomegranate juice concentrate reduced the volume and weight of xenografted BT-474 tumors in female athymic nude mice [26]. 

Recently, we have documented the novel finding that oral feeding of a pomegranate emulsion (PE), containing most bioactive phytochemicals present in the whole fruit, exerted a significant chemopreventive activity against DMBA-initiated mammary tumorigenesis in female rats [34]. PE reduced the incidence, total burden and average weight of mammary tumors with a concomitant inhibition of intratumor cell proliferation, induction of apoptosis, and it altered the expression of Bax, Bcl2, Bad, caspase-3, caspase-7, caspase-9, poly (ADP ribose) polymerase and cytochrome *c* [34]. We have also observed that PE diminished the expression of estrogen receptor-α (ER-α), ER-β and cyclin D1 and abrogated the expression, cytoplasmic accumulation and nuclear translocation of β-catenin, an essential transcriptional cofactor for Wnt/β-catenin signaling, during DMBA mammary carcinogenesis [35]. Emerging studies indicate that chronic inflammation is involved in the development and progression of mammary carcinoma [36,37,38,39,40] and pomegranate phytochemicals are endowed with anti-inflammatory properties [4,8,11,12]. Accordingly, this study was conducted to investigate the anti-inflammatory mechanisms of PE administration by analyzing various proinflammatory and stress markers, such as cyclooxygenase-2 (COX-2) and heat shock protein 90 (HSP90) as well as several inflammation-regulatory pathways, namely nuclear factor-κB (NF-κB) and nuclear factor erythroid 2p45 (NF-E2)-related factor 2 (Nrf2) signaling, during DMBA-inflicted mammary gland tumorigenesis in rats.

## 2. Materials and Methods

### 2.1. Test Materials, Chemicals and Antibodies

PE, a proprietary formulation containing pomegranate aqueous extract and seed oil, was purchased from Rimonest Ltd. (Haifa, Israel). A detailed description of the preparation of this emulsion has been published previously [15]. The chemical analyses of this formulation revealed the presence of caffeic acid, corilagin, ellagic acid, ferulic acid, gallic acid, 5-hydroxymethylfurfural, protocatechuic acid, punicalagins (A and B) and *trans*-p-coumaric acid in the aqueous phase and mixed octadecatrienoic acids, sterols and steroids (e.g., 17-α-estradiol), tocol and γ-tocopherol in the lipid phase [15]. The mammary carcinogen DMBA was procured from Sigma-Aldrich (St. Louis, MO, USA). Paraformaldehyde was obtained from Ted Pella (Redding, CA, USA). Primary antibodies, such as COX-2, inhibitory κBα (IκBα), NF-κB-p65, Nrf2 as well as the ABC staining kit were purchased from Santa Cruz Biotechnology (Santa Cruz, CA, USA). HSP90 was a product of Enzo Life Sciences (Farmingale, NY, USA). 

### 2.2. Experimental Protocol and Tumor Tissue Harvesting

Breast tumor sections for this work were collected from our previously completed chemopreventive study [34] based on an animal protocol approved by the Institutional Animal Care and Use Committee of Northeast Ohio Medical University (Rootstown, OH, USA). In short, female Sprague-Dawley rats (Harlan Laboratories, Indianapolis, IN, USA) were divided into six separate groups. All animals were fed a basal diet (LabDiet, St. Louis, MO, USA) ad libitum. Group A (*n* = 12) and group B (*n* = 11) were kept untreated. The remaining rats were orally administered (gavaged) with PE three times per week as follow: 0.2 g/kg (group C, *n* = 8), 1.0 g/kg (group D, *n* = 8) and 5.0 g/kg (group E, *n* = 7 and group F, *n* = 5) for a total of 18 weeks. Two weeks following the commencement of the study, animals from groups B, C, D and E were orally administered with a single dose of DMBA (50 mg/kg body weight) to induce mammary tumorigenesis. Eighteen weeks after initiation of the study, all animals were sacrificed and mammary glands were harvested and fixed in 4% paraformaldehyde for further analysis. 

### 2.3. Immunohistochemical Analysis

The intratumor protein expressions of COX-2, HSP90, IκBα, NF-κB-p65 and Nrf2 were analyzed by methods described previously [41]. In brief, tumor tissue sections were first hydrated using phosphate-buffered saline followed by incubation in sodium citrate buffer for antigen retrieval. Endogenous peroxidases were blocked by treating the sections with 1% H_2_O_2_. Tissue sections were then treated with blocking solution followed by washing with phosphate-buffered saline and incubating overnight at 4 °C with primary antibodies (1:100 dilution). After several washes, tissue sections were treated with horseradish peroxidase-conjugated secondary antibody (1:200 dilution) for 30 min at room temperature, and then with 3,3′-diaminobenzidine tetrahydrocholoride solution to visualize brown antigen-antibody complexes. Finally, the sections were counterstained with Gill’s hematoxylin solution. The immunohistochemical slides were viewed under a light microscope (BX43, Olympus, Center Valley, PA, USA) and 1000 tumor cells/rat were analyzed. 

### 2.4. Statistical Analyses

All data are expressed as mean ± standard error of mean (SEM). Statistical analyses were performed by using a commercial software (SigmaPlot 11.0, Systat Software, Inc., San Jose, CA, USA). One-way analysis of variance with least significant difference post hoc analysis was employed to compare various parameters among different treatment and control groups. A *p*‑value less than 0.05 was considered statistically significant. 

## 3. Results

### 3.1. PE Abrogated Elevated COX-2 Expression during DMBA-Induced Mammary Tumorigenesis

**Figure 1:**
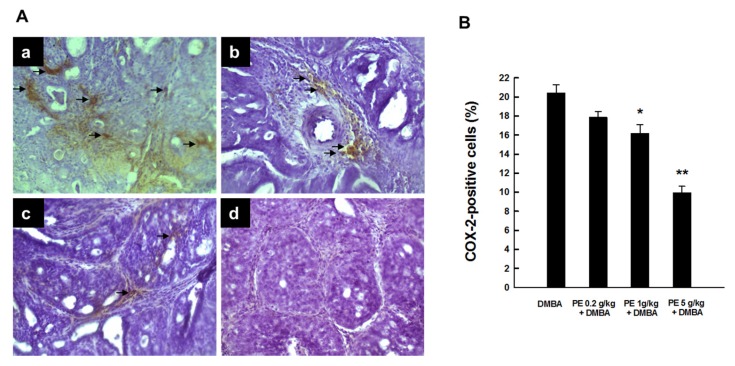
COX-2 expression during 7,12-dimethyl benz(a)anthracene (DMBA)-induced breast tumorigenesis in rats in the presence or absence of pomegranate emulsion (PE) treatment. (**A**) Immunohistochemical localization of COX‑2-positive cells (arrows) in tumor sections (magnification: ×200). The various treatment groups are: (**a**) DMBA control; (**b**) PE (0.2 g/kg) plus DMBA; (**c**) PE (1 g/kg) plus DMBA; and (**d**) PE (5 g/kg) plus DMBA. (**B**) Quantitative analysis of COX-2-immunopositive cells from representative images. Results (mean ± SEM) are based on 1000 cells per animal and four animals per group. * *p* < 0.01 and ** *p* < 0.001 compared to the DMBA control.

A substantial expression of COX-2 was observed predominantly in the cytoplasm of tumor cells of DMBA control animals (Figure 1(Aa)). PE at a dose of 0.2 g/kg slightly reduced intratumor COX-2 immunopositivity compared to the DMBA control (Figure 1(Ab)). On the other hand, a moderate and drastic inhibition of COX-2 expression was noticed following PE treatment at a dose of 1 and 5 g/kg, respectively (Figure 1(Ac) and Figure 1(Ad)). Although PE at 0.2 g/kg slightly decreased the percentage of COX-2-positive cells, this result was statistically insignificant (Figure 1B). On the other hand, there was a significant (*p* < 0.01 or 0.001) inhibition of the percentage of intratumor COX-2-positive cells in animals administered with 1 or 5 g/kg PE compared to the DMBA control, respectively. 

### 3.2. PE Suppressed HSP90 Expression during DMBA Mammary Tumorigenesis

**Figure 2:**
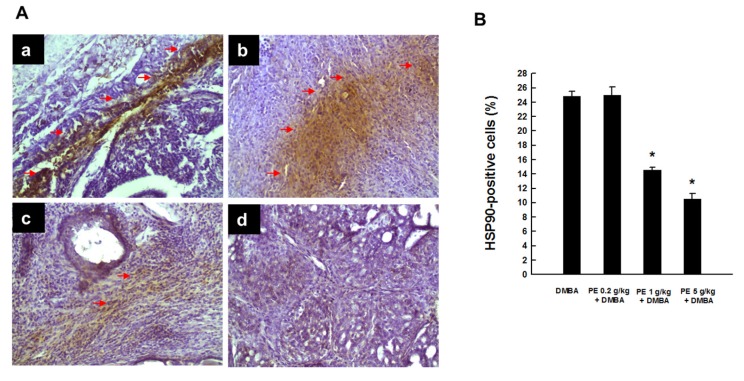
HSP90 expression during DMBA-induced breast tumorigenesis in rats in the presence or absence of PE treatment. (**A**) Immunohistochemical localization of HSP90-positive cells (arrows) in tumor sections (magnification: ×200). The various treatment groups are: (**a**) DMBA control; (**b**) PE (0.2 g/kg) plus DMBA; (**c**) PE (1 g/kg) plus DMBA; and (**d**) PE (5 g/kg) plus DMBA. (**B**) Quantitative analysis of HSP90-immunopositive cells from representative images. Results (mean ± SEM) are based on 1000 cells per animal and four animals per group. * *p* < 0.001 compared to the DMBA control.

Tumor sections from the DMBA control animals showed a considerable expression of HSP90 (Figure 2(Aa)). PE treatment at 0.2 g/kg in conjunction with DMBA exposure did not change intratumor HSP90 expression (Figure 2(Ab)). PE at 1 g/kg substantially reduced the expression of HSP90 in tumor samples compared to the DMBA control (Figure 2(Ac)). A further decrease of HSP90 was achieved with PE at 5 g/kg (Figure 2(Ad)). The quantitative data on HSP90-immunopositivity revealed dose-dependent and statistically significant (*p* < 0.001) suppression of this protein expression in tumor samples from DMBA-treated rats that received PE treatment at 1 or 5 g/kg (Figure 2B). 

### 3.3. PE Inhibited Activation of NF-κB during DMBA Mammary Tumorigenesis

**Figure 3:**
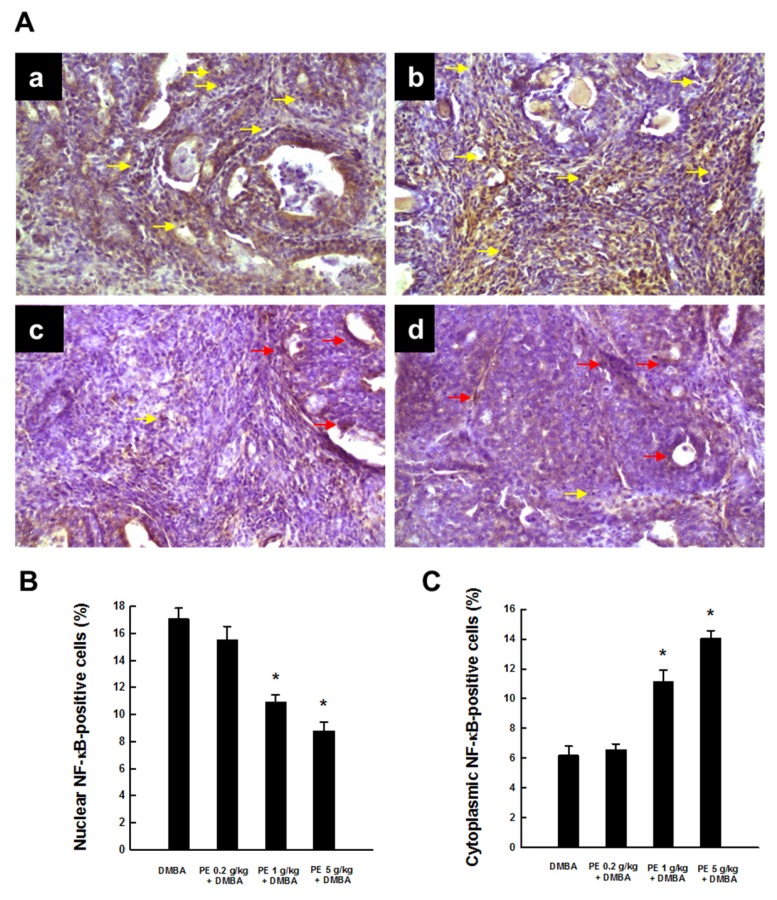
NF-κB p65 expression during DMBA-induced breast tumorigenesis in rats in the presence or absence of PE treatment. (**A**) Immunohistochemical localization of NF-κB p65 in nucleus (yellow arrows) and cytoplasm (red arrows) (magnification: ×200). The various treatment groups are: (**a**) DMBA control; (**b**) PE (0.2 g/kg) plus DMBA; (**c**) PE (1 g/kg) plus DMBA; and (**d**) PE (5 g/kg) plus DMBA. Quantitative analysis of (**B**) nuclear and (**C**) cytoplasmic NF-κB-immunopositive cells from representative images. Results (mean ± SEM) are based on 1000 cells per animal and four animals per group. * *p* < 0.001 compared to the DMBA control.

We observed an extensive expression of NF-κB p65 in the nucleus and very limited expression of this protein in the cytoplasm in tumor sections harvested from the DMBA control animals, suggesting activation and subsequent translocation of NF-κB p65 from cytosol to nucleus (Figure 3(Aa)). Almost similar expression of nuclear and cytoplasmic NF-κB p65 was noticed following the treatment with PE at 0.2 g/kg (Figure 3(Ab)). Conversely, PE at 1 or 5 g/kg attenuated nuclear NF-κB p65 expression and elevated the expression of this protein in cytosol (Figure 3(Ac) and Figure 3(Ad), respectively). The quantitative analysis of NF-κB p65-immunopositive cells displays a significant (*p* < 0.001) decrease in nuclear NF-κB p65-positive cells (Figure 3B) and a significant (*p* < 0.001) increase in cytoplasmic NF-κB p65-positive cells (Figure 3C) in two PE-treated groups (1 and 5 g/kg) compared to the DMBA control. 

**Figure 4:**
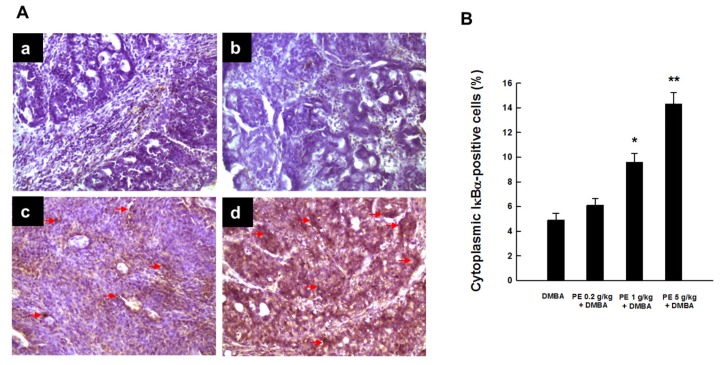
IκBα expression during DMBA-induced breast tumorigenesis in rats in the presence or absence of PE treatment. (**A**) Immunohistochemical localization of IκBα-positive cells (arrows) in the cytoplasm of tumor sections (magnification: ×200). The various treatment groups are: (**a**) DMBA control; (**b**) PE (0.2 g/kg) plus DMBA; (**c**) PE (1 g/kg) plus DMBA; and (**d**) PE (5 g/kg) plus DMBA. (**B**) Quantitative analysis of IκBα-immunopositive cells from representative images (**A**). Results (mean ± SEM) are based on 1000 cells per animal and four animals per group. * *p* < 0.01 and ** *p* < 0.001 compared to the DMBA control.

We also noticed very limited expression of cytosolic IκBα in the DMBA control animals (Figure 4(Aa)), indicating the possible degradation of IκBα protein. PE treatment inhibited DMBA-induced degradation of IκBα protein in cytosol in a dose-dependent fashion. Our results showed a marginal increase in cytosolic IκBα expression by 0.2 g/kg PE (Figure 4(Ab)) and sizable upregulation of this protein by 1 or 5 g/kg PE (Figure 4(Ac,Ad), respectively). These results are supported by the quantitative analysis of IκBα-positive cells that demonstrated a significant (*p* < 0.01 or 0.001) increase of immuno-positive cells in the mammary tumor sections harvested from rats treated with 1 or 5 mg/kg PE, respectively (Figure 4B). 

### 3.4. PE Induced Nrf2 Expression during DMBA Mammary Tumorigenesis

**Figure 5:**
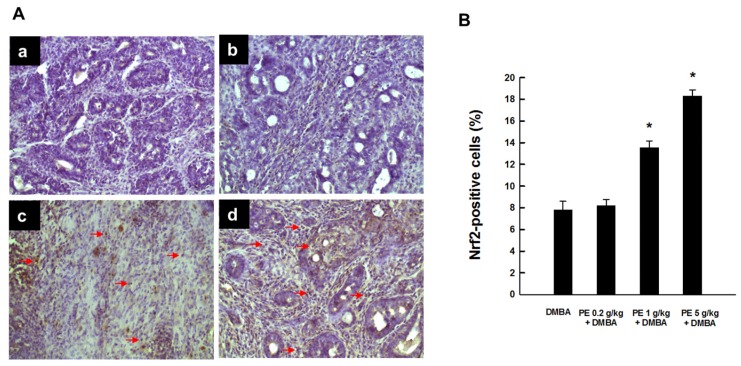
Expression of Nrf2 from DMBA-induced breast tumors in rats treated with or without PE. (**A**) Immunohistochemical localization of Nrf2-positive cells (arrows) in the nucleus of tumor sections (magnification: ×200). The treatment groups are: (**a**) DMBA control; (**b**) PE (0.2 g/kg) plus DMBA; (**c**) PE (1 g/kg) plus DMBA; and (**d**) PE (5 g/kg) plus DMBA. (**B**) Quantitative analysis of Nrf2-immunopositive cells from representative images. Results (mean ± SEM) are based on 1000 cells per animal and four animals per group. * *p* < 0.001 compared to the DMBA control.

Figure 5A shows the intratumor immunohistochemical profiles of Nrf2 expression of various animal groups. The DMBA control group exhibited a marginal expression of Nrf2 (Figure 5(Aa)). PE treatment at 0.2 g/kg showed a similar expression of Nrf2 (Figure 5(Ab)). In contrast, tumor sections from DMBA-exposed animals treated with 1 or 5 g/kg PE showed a strong upregulation of Nrf2 (Figure 5(Ac,Ad), respectively). The majority of Nrf2-immunopositivity was noticed in the nucleus, suggesting the activation and subsequent translocation of Nrf2 from the cytoplasm to the nucleus. Our quantitative analysis revealed a statistically significant (*p* < 0.001) increase in the percentage of Nrf2-positive cells following PE treatment at 1 or 5 g/kg compared to the DMBA control (Figure 5B)

## 4. Discussion

Breast cancer is the second leading cause of death in women worldwide. Globally, more than one million women are diagnosed with breast tumor. In the United States, breast cancer is the second most frequent female cancer. Approximately 246,660 new cases of breast cancer and 40,450 breast cancer-related deaths were estimated to occur in women in the United States in 2016 [42]. Emerging evidence suggests that chronic inflammation contributes to breast cancer development and progression [36,37,38,39,40]. Experimental studies have provided convincing evidence that various inflammatory molecules and signaling pathways are involved in the proliferation, survival, epithelial-mesenchymal transition, invasion and metastasis of breast cancer cells [43]. Accordingly, agents that inhibit chronic inflammation may be effective in the prevention and therapy of mammary carcinoma. Numerous natural products, phytochemicals and dietary agents with anti-inflammatory activities have shown promise in the prevention and treatment of breast cancer [44,45,46,47,48,49]. Recently, we have published the novel finding that a new formulation (PE) consisting of pomegranate phytoconstituents exerts a striking chemoprevention of DMBA-initiated rat mammary tumorigenesis though the molecular mechanisms of action of such beneficial activity is not completely elucidated [34]. Accordingly, the present work was designed to investigate the ability of PE to interfere with DMBA-mediated inflammatory signaling cascades by analyzing breast tumor sections harvested from our earlier study [34].

The COX family of enzymes catalyzes the rate-limiting step in the synthesis of prostaglandins (PGs), including PGE2, mainly from arachidonic acid. There are two isoforms of COX enzymes, namely COX-1 and COX-2. COX-1 is constitutively expressed in most cells, whereas the expression of COX-2 is induced by various stimuli, including shear stress, cytokines, growth factors and oncogenes [50]. PGs produced by COX-2 are involved in various critical steps in oncogenesis, including proliferation, mutagenesis, apoptosis evasion, immune suppression, angiogenesis and invasion [51,52]. Interestingly, the inhibition of COX-2 has been shown to reduce breast tumor cell proliferation [53,54]. COX-2 protein expression in ductal carcinoma in situ and invasive breast carcinoma indicates the crucial role of COX-2 in early stages of mammary carcinogenesis [55]. Our results clearly indicate the PE-mediated suppression of COX-2 in chemically-induced mammary carcinogenesis in rats. We suggest that our previously reported mammary tumor inhibitory effect of PE in DMBA carcinogenesis [34] could be, at least in part, due to the inhibition of COX-2 expression. Other investigators reported that cold-pressed pomegranate seed oil inhibited sheep cyclooxygenases by around 30–40% [56]. In addition, plasma isolated from rabbits following oral ingestion of pomegranate fruit extract inhibited both COX-1 and COX-2 enzymes ex vivo, and the effect was more pronounced for COX-2 [57]. Finally, several pomegranate constituents, e.g., ellagic acid, gallic acid and punicalagin A and B, inhibited lipopolysaccharide-induced PGE2, nitric oxide and interleukin-16 (IL-6) production [58].

Heat shock proteins (HSPs) represent stress-inducible proteins which are known to play important roles in cellular stress response. There are several core HSP families in humans, such as DNAJ (HSP40), HSPA (HSP70), HSPB (small HSP), HSPC (HSP90), HSPD/E (HSP60/HSP10), HSPH (HSP110) and CCT (TRiC). HSP90, a highly conserved and abundant 90 kDa protein, is involved in chaperoning the structures of over 200 client proteins, many of which are involved in growth control as well as mammary tumor cell proliferation [59]. Based on scientific reports, HSP90 may regulate inflammatory events through the modulation of various cytokines and cell signaling pathways [60,61,62]. The efficacy of various HSP90 inhibitors in the treatment of several oncologic diseases, including breast cancer, is currently underway [63,64,65]. In our study, an elevated expression of HSP90 in tumor sections suggests that DMBA may exert heat shock response, perhaps due to inflammatory stress, leading to the irregular proliferation and evasion of apoptosis, as we have previously observed [34]. Our present results show the substantial reduction of HSP90 expression in the PE plus DMBA group, suggesting the capability of PE to abrogate mammary tumor cell growth and survival by the downregulation of HSP90, which may be associated with a lesser magnitude of inflammatory stress. A recent study shows that pomegranate exerts anti-inflammatory effects by upregulating HSP70, transforming growth factor‑β1 (TGF-β1) and IL-10 expression during chemically-induced hepatotoxicity in rats [66].

Various signaling pathways activated in oncogenesis are likely to be networked through the activation of NF-κB, a proinflammatory transcription factor [67]. The major inactive form of NF-κB complex is a p50–p65 heterodimer which binds to the inhibitory protein IκBα and predominantly resides in the cytoplasm. In the classical (canonical) pathway initiated by proinflammatory cytokines, such as tumor necrosis factor-α and IL-1β, IκBα undergoes degradation by IκB kinase (IKK)γ-containing IKK complex via the TGF-β1-dependent pathway [68]. Subsequently, the free p50–p65 dimer translocates into the nucleus, binds to its cognate response element in the DNA, and induces the transcription of a battery of target genes involved in proliferation, survival, inflammation, angiogenesis, invasion and metastasis [69,70]. A critical association between NF-κB-mediated inflammatory pathways and breast cancer development and progression has been well established [37,71]. Abrogation of constitutive activation of NF-κB diminishes the oncogenic potential of transformed cells, and the suppression of this proinflammatory pathway may be valuable for cancer prevention and therapy [72,73,74]. In our present study, the limited expression of NF-κB p65 and IκBα in cytosol and the substantial expression of NF-κB p65 in the nucleus of mammary tumor cells from animals exposed to DMBA only indicate the conceivable degradation of IκBα, release of activated NF-κB p65, and its subsequent translocation to the nucleus. These results are supported by an earlier study which documented that the activation of NF-κB is implicated in DMBA-initiated early malignant transformation in rat mammary glands [75]. Our results of PE-mediated protection against IκBα degradation and hindrance with the translocation of activated NF-κB p65 to the nucleus suggest that PE possibly impedes early events in DMBA-induced mammary carcinogenesis in rats. Consistent with our results, Khan and colleagues [21] observed that pomegranate fruit extract inhibited constitutive NF-κB activation and NF-κB-dependent reported gene expression associated with proliferation, invasion and motility in two aggressive breast carcinoma cells, namely MDA-MB-231 and SUM 149. Banerjee and coinvestigators [26] also reported that pomegranate juice concentrate suppressed the growth of MDA-MB-231 cells concomitant with reduced transcriptional and translational expression of NF-κB. Similar results were observed in nude mice xenografted with BT474 breast carcinoma cells [26].

Nrf2, a basic-leucine zipper transcription factor, is known to play a critical role in protecting mammalian cells against injury inflicted by inflammation and oxidative stress [76]. Under normal cellular conditions, Nrf2 is anchored in the cytoplasm by its association with Kelch-like erythroid Cap-N-Collar homolog-associated protein 1 (Keap1). Upon cellular stress as well as pharmacological stimuli, Nrf2 is liberated from Keap1, translocates to the nucleus, recognizes and binds to *cis*-acting enhancer known as an antioxidant response element or electrophile response element, and eventually facilitates the transcription of a plethora of genes that encode antioxidant and detoxifying enzymes [77]. The Nrf2-mediated signaling pathway is profoundly involved in the regulation of inflammation and inflammation-associated carcinogenesis, and accordingly this pathway embodies an important target for the prevention of inflammation-related cancer [78,79,80]. Interestingly, a potential cross-talk between Nrf2 and NF-κB transcription factors modulated by mitogen-activated protein kinase to influence inflammation-associated etiopathogenesis of cancer has been proposed [81,82]. Singh and colleague [83,84,85] elegantly showed decreased protein and mRNA expression of Nrf2 and Nrf2-regulated genes in estrogen-exposed mammary tissue and mammary tumors in rats. In line with the aforementioned results, we have also found the limited expression of Nrf2 in rat mammary tumors induced by DMBA. However, PE treatment was effective in upregulating the expression as well as the nuclear translocation of Nrf2 which may, in turn, relieve NF-κB-mediated inflammatory action and ultimately lead to breast cancer prevention. However, it is noteworthy to mention another aspect of Nfr2 induction. Stable expression of Nfr2 results in the enhanced resistance of cancer cells to chemotherapeutic agents, including cisplatin, doxorubicin and etoposide. The increment of Nrf2, induced by PE, can be useful for the prevention of inflammation associated with carcinogenesis, but it can compromise the therapeutic outcome of patients treated with chemotherapeutic drugs.

The identification of specific phytochemicals of PE responsible for the observed anti-inflammatory activities during experimental mammary tumorigenesis may not be possible at this time, and requires further investigations. Phytochemical profiling of pomegranate reveals the presence of several constituents, including ellagic acid, ellagitannins, punicic acid, and punicalagin, which are capable of the modulation of inflammatory cell signaling [86,87,88]. In view of the notion that pomegranate phytochemicals exhibit maximum beneficial effects when they are used in combination rather than individually [89], it is reasonable to propose that various phytochemicals present in this unique fruit may regulate various inflammatory molecules and cascades during mammary carcinogenesis through a synergistic effect. 

## 5. Conclusions

The present results coupled with those we previously reported possibly indicate that the prevention of DMBA-initiated mammary tumorigenesis by PE may involve anti-inflammatory mechanisms propelled by synchronized and differential regulation of two interrelated molecular pathways, such as NF-κB and Nrf2 signaling. Further in-depth studies using appropriate techniques are required to confirm the results presented here, in order to facilitate the development of a pomegranate emulsion for the prevention and treatment of cancers linked to inflammation.
